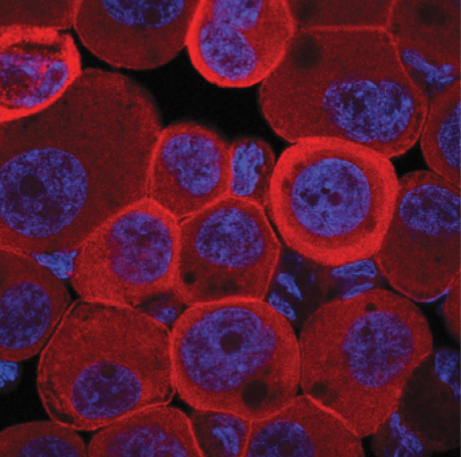# Elucidating metastasis mechanisms in lobular breast cancer

**Published:** 2015-04

To metastasise, cancer cells need to escape anoikis – apoptosis induced by loss of cell-matrix adhesion. Invasive lobular cancer (ILC) is a major breast cancer type that is particularly prone to metastasise owing to its intrinsic anoikis resistance and infiltrative growth pattern. Current treatments for breast cancer are often ineffective for metastatic ILC. To understand the basis of anoikis-resistance mechanisms in this disease, Patrick Derksen’s group used mouse ILC models and performed genome-wide mRNA profiling. They found that, in ILC cells, anchorage independency is mediated by the upregulation of *Wnt11* (a noncanonical Wnt signal), which, in turn, is controlled by the nuclear translocation of p120-catenin (a protein involved in cell-cell adhesion processes) through inhibition of Kaiso-mediated transcriptional repression. In addition, *Wnt11* promotes activation of RhoA – a member of the Rho-Rock pathway that drives ILC anoikis resistance and subsequent tumour growth and metastasis. This study identifies a p120/Kaiso/Wnt11-dependent autocrine RhoA activation loop that drives anchorage independence and underpins the use of Rho-Rock targeting as a therapeutic strategy in the treatment of metastatic ILC. **Page 373**

**Figure:**